# Evolving requirements for materials modelling software and underlying method developments: an inventory and future outlook

**DOI:** 10.12688/openreseurope.14843.2

**Published:** 2022-08-01

**Authors:** Ilian Todorov

**Affiliations:** 1 Scientific Computing Department, Science and Technology Facilities Council, Warrington, Cheshire, WA4 4AD, UK

**Keywords:** materials modelling, molecular simulations, method development, scientific software, computer simualtions, HPC

## Abstract

This European Materials Modelling Council (EMMC) study provides an outline of the survey intent and ambitions, followed by an analysis of the results and a follow up discussion, focused on the future perspectives of the EMMC. The survey covers materials modelling and characterisation communities in both academia and industry. It provides a profile of the surveyed players in these communities and a scaled measure on their usage of computational methodologies. The survey outcomes include: (i) summary views of the recent as well as perceived future trends of materials modelling and its associated fields, with respect to two focus areas surveyed, Model Development and Software, (ii) the main adoption factors and associated bottlenecks for computational methods and software, (iii) the most targeted materials properties and digital twins approaches, and (iv) the wider communities expectations of how EMMC can help facilitate, fulfil and drive further the European Materials Modelling Roadmap to the benefit of the European Commission’s (ECs’) research and innovation.

## Plain language summary

The European Materials Modelling Council (EMMC) focus areas of Model Development and Software lead the EMMC’s activities in their domains and the key needs of their representative researcher communities are summarised as follows. For Software these are more accurate, robust, well-documented and validated/verified software, with better availability of parameters data (easy generation of input), applicability (general purpose/demonstrators), scalable performance & lower complexity to use. For Model Development these are improved capability, accessibility and performance of methods, with better applicability (general purpose over specificity).

This survey endorsed the point that digital tools drive the future of materials in chemical and manufacturing industries, by providing agility and speed in the development process. The materials digitalisation wave relies on using physics-based modelling approaches as well as data-driven approaches and their use together, to accurately predict and optimise industrial products in an early design stage. The materials research community, including methodology developers and software owners, provides cutting edge materials modelling methods and software that both require continued support by funding bodies and organisations, nationally and internationally.

## Introduction

This report is based on the kick-off survey initiative, instigated by the Software and Model Development focus areas (FAs) of the European Materials Modelling Council (EMMC) upon re-establishing the council from a H2020 coordinated support action project and association into a professional community as a non-profit organisation ( *association sans but lucratif* [ASBL]). The two FAs made a fresh *ad hoc* start in May 2020 and were interested in better identifying their perspective communities and their needs and expectations in order to drive their own development initiatives and help develop EMMC ASBL as a bottom-up organisation. Ilian Todorov, acting as co-chair of the EMMC Organisational Assembly and of the Software FA, proposed that a common survey from the two FAs “ *is of benefit to both as they are ultimately linked, connected and bound by the subject of scientific methodology*”. Despite the large commonality and overlap of these two FAs’ audiences, their main stakeholder groups are very different and can be summed as a *modelling and simulation researchers, modellers and theorists/theoreticians* for the Model Development FA; and *software owners, computational scientists, computer scientists and research software engineers* for the Software FA. It is worth noting that both FAs include *data scientists* and *experimentalists* (characterisation) who are also intrinsically associated with the stakeholder roles described above.

The survey was carried out between 21 November 2020 and 12 January 2021, as commissioned and endorsed by EMMC. Its main purpose was to map the landscape of materials modelling by (i) collecting a focused feedback from the wider community of materials modellers with respect to the two FAs (a cross-section of many communities that work in these areas or benefit from them directly) and (ii) identifying and clarifying the communities’ interests for the benefit of the EMMC. The survey outcomes are summarised in the following sections and give a clear mandate to the FA chairs as well as to the EMMC board of directors (BoD) to act upon these.

## Methods

This survey was the very first one organised on behalf of the EMMC ASBL. As such it aimed to classify and identify the participating audience, their interest with respect to the EMMC main areas of business and their background. This classification included identification of funding support and models of operation of the participants’ hosting institutions. The small size of the survey was deliberately chosen in order to keep participants focused. Several questions on identification, strengths and weaknesses offered a close preselected choice of options in order to produce compact and easy to identify and analyse trends. However, many questions were offered in an open format and concerned the wide spread of EMMC terminology and methodologies, e.g., the MOdelling DAta (MODA) ( de Baas, 2017).

Participation was invited via multiple communities’ mailing lists ( CCP5 [Computational Collaboration Project No 5], HEC-MCC [High End Computing for the Materials Chemistry Consortium], CCPBioSim [Collaborative Computational Project for Biomolecular Simulation], HEC-BioSim [High End Computing for the Biomolecular Simulation Community], CCP4 [Computational Collaboration Project No 4], CCP9 [Computational Collaboration Project No 9], Psi-k – *Ab initio* (from electronic structure) calculation of complex processes in materials Network, MOLECULAR-DYNAMICS-NEWS @ JISC, EMMC) and social platforms such as LinkedIn and Twitter.

The participants were made aware of EMMC and EMMC ASBL ambitions and were provided with a link to identify the relevant FAs and their description. The Model Development FA was defined as: ‘Everything that has to do with the capabilities and qualities of the materials models and the modelling workflows: development, validation and application. *’* The Software FA was defined as: ‘Successful materials modelling software uses the best algorithms, it is numerically robust, carefully validated, well documented, easy to use, and continuously maintained and supported over decades.’. The survey was conducted by a web-form, the collected data from which were anonymised and subsequently analysed as described below.

## Results

The following sections summarise the results of the survey ( Todorov, 2022), which had 98 respondents in total.

### The players

**Figure 1.**
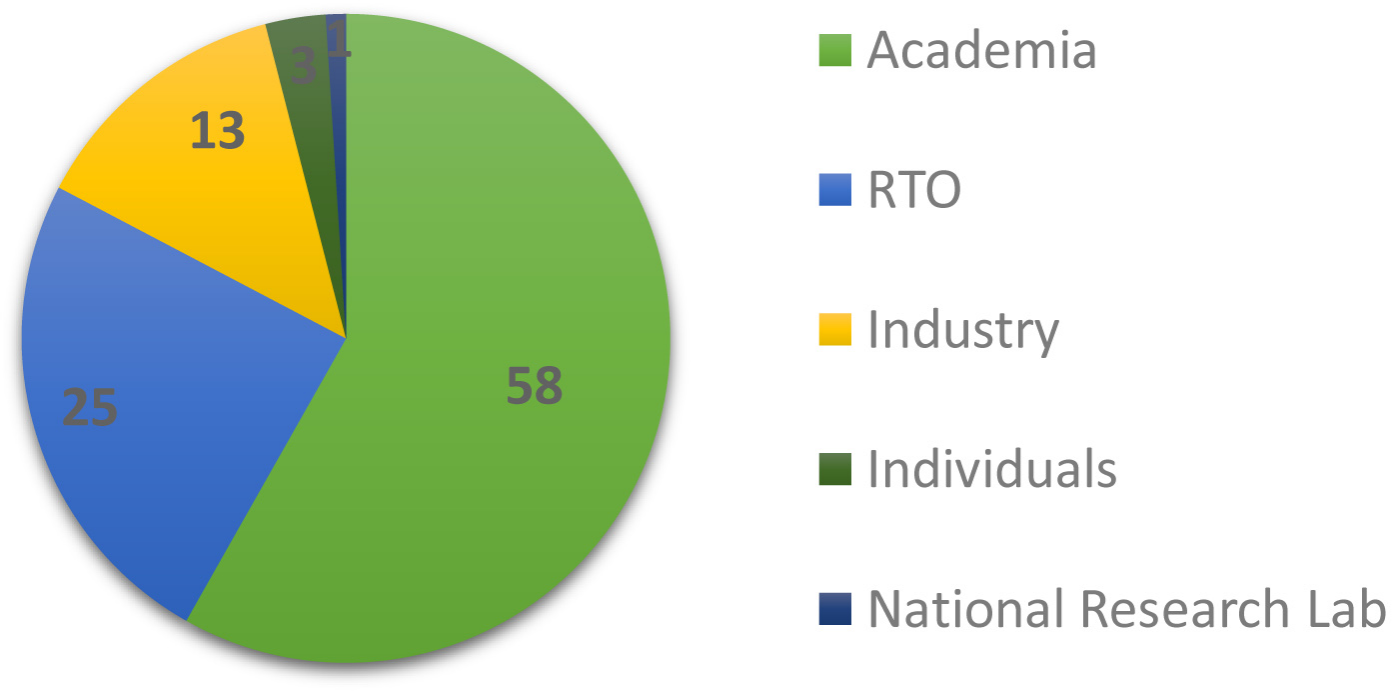
AFFILIATION OF RESPONDENTS IN % (RTO - RESEARCH AND TECHNOLOGY ORGANISATION).

This section is concerned with the background and composition of the respondents. Approximately 46% of them were affiliated with the EMMC ASBL as members. As depicted in Figure 1, 58.2% were based in academia (universities/higher education), 24.5% in research and technology organisations (RTOs) and 13.3% in industry; 3% answered as individuals and only ~1% were affiliated with national research laboratories.

**Figure 2.**
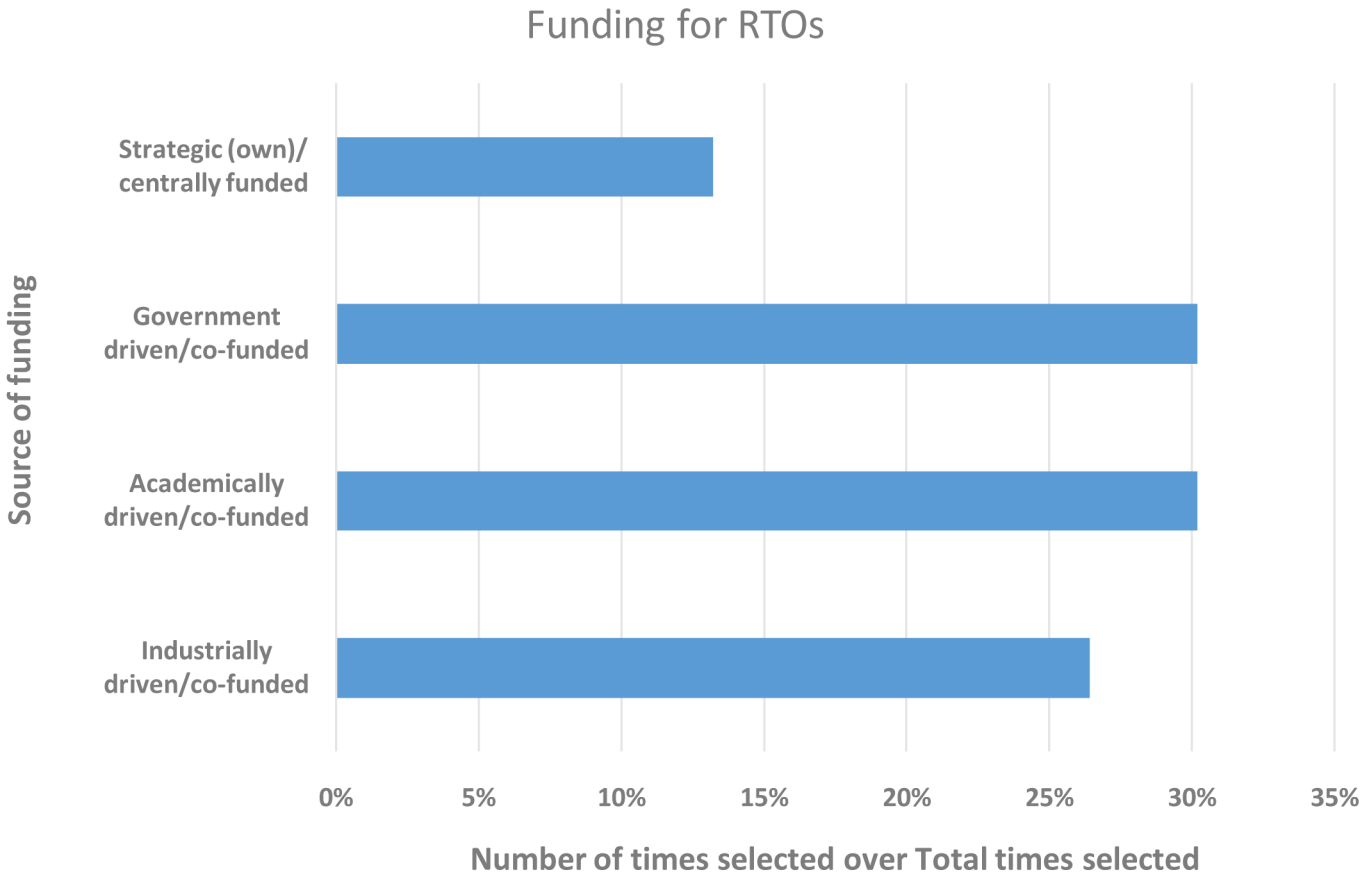
FUNDING FOR RESEARCH AND TECHNOLOGY ORGANISATIONS (RTOS) WITH THE Y-AXIS SHOWING THE SOURCE OF FUNDING AND THE X-AXIS THE NUMBER OF TIMES A FUNDING SOURCE WAS SELECTED. THE 27 RESPONDENTS WERE ALLOWED TO SELECT MORE THAN ONE SOURCE.

RTOs ( Charles & Ciampi Stancova, 2015) have been established in many European countries with the aim of assisting local industry around a specific set of industrial technologies or focusing on specific industrial sectors to advance their development and digitalisation. They are often founded with governmental funding and are either planned to self-sustain in the long-term or are strongly encouraged to diversify within a few years and develop opportunities to rely more on non-government funding from local and international sources, e.g., industry or EC research and innovation (R&I) funding calls. Hence, we took an inventory of all respondents from RTOs to gain an understanding of how their funding situation unfolded at this very time (from 15 December 2020 to 15 January 2021), since the uptake of materials modelling would always require some initial monetary input. In total, 27.7% of the respondents qualified for this additional survey and were given the option to choose more than one funding source, if and as applicable. In Figure 2, one can see that the RTOs had similar amounts of funding from government, academia and industry, while strategic (own) funding streams were emerging.

**Figure 3.**
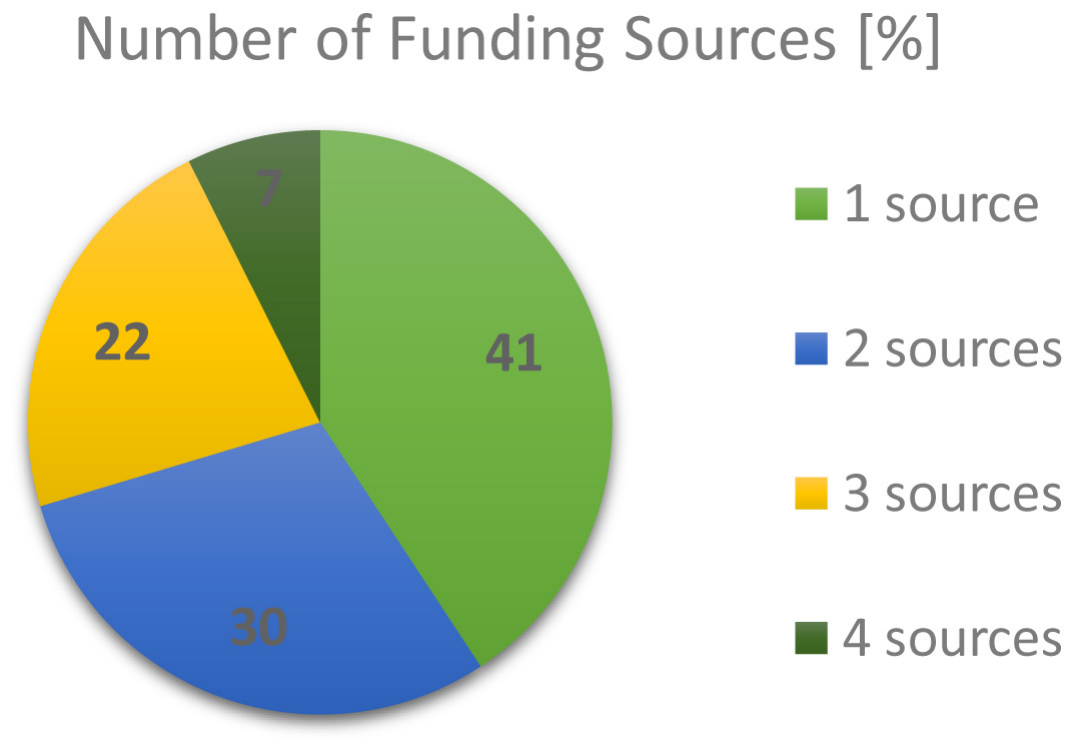
NUMBER OF FUNDING SOURCES IN % CLAIMED BY RESEARCH AND TECHNOLOGY ORGANISATIONS (RTOS) SIMULTANEOUSLY.

A closer look into the funding landscape outcomes from the RTO respondents, as depicted in Figure 3, reveals that more often a mixture of several types of funding was relied upon than just a single type. Overall 40.7% claimed their funding from one source, 29.6% from two sources, 22.2% from three sources and 7.4% from four sources. Approximately 60% of all RTO respondents still relied on some amount of government funding, which was on par with funding coming from research councils (academic in nature). However, the funding landscape clearly showed, with ~50% industrial co-funding, that the private sector played a significant role in the business model of RTOs.

Of the 13 industrial participants, nine identified themselves as being from the software and services domain (~70%), two were from materials manufacturing companies (~15%) and one each were from the chemical and the oil and gas industries.

The respondents were asked to share which roles they take on (multiple choice of roles was allowed) in their respective organisations, which are shown in Table 1.

**Table 1.** T he roles a respondent takes on in their organisation.

Expertise/roles	Abbreviation	Number of times selected	%
**Research software developer ( Robert Baxter *et al.*, 2012)**	RSE	33	17.3
**Researcher/modeller developing models/workflows**	ResDev	49	25.7
**Researcher/modeller (using software for producing research data)**	ResUser	59	30.9
**Digitalisation and Interoperability practitioner** - semantic workflows ( Horsch *et al.*, 2020) (ontologies [ Goldbeck *et al.*, 2019; Horsch, 2020], metadata, cloud integration), code coupling for multi-physics and multiscale	InterOp	10	5.2
**Translator/consultant**	Trans	12	6.3
**Business and innovation developer** (sales & marketing)	BID	5	2.6
**Software distributor**	SoftDist	0	0.0
**Coordinator** (manager/administrator of project/programme/team)	Manager	23	12.0
**Total times selected**		**191**	

**Figure 4.**
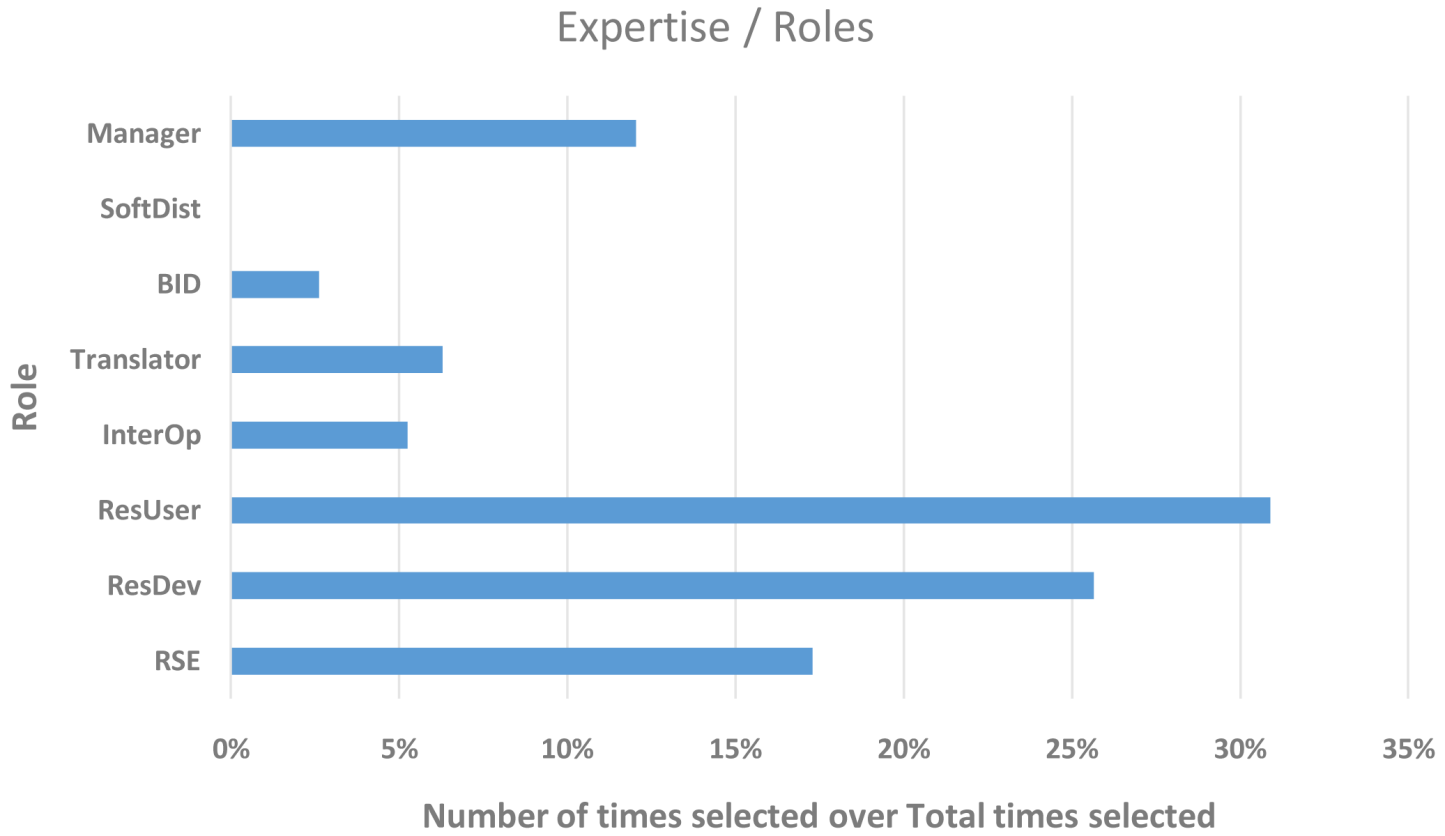
EXPERTISE/ROLES OF THE RESPONDENTS WITH THE Y-AXIS SHOWING THE ROLE AND THE X-AXIS THE NUMBER OF TIMES A ROLE WAS SELECTED. THE 98 RESPONDENTS WERE EACH ALLOWED TO SELECT MORE THAN ONE ROLE (RSE - RESEARCH SOFTWARE DEVELOPER, RESDEV – RESEARCHER/MODELLER DEVELOPING MODELS/WORKFLOWS, RESUSER – RESEARCHER/MODELLER [USING SOFTWARE FOR PRODUCING RESEARCH DATA], INTEROP - DIGITALISATION AND INTEROPERABILITY PRACTITIONER, BID – BUSINESS AND INNOVATION DEVELOPER, SOFTDIST – SOFTWARE DISTRIBUTOR).

Figure 4 shows that the majority of our respondents were using or developing software (~56%) and 23 of them were in a managerial role (~12% of the total responses). A few individuals (~5% of the total) were practising digitalisation and interoperability, similar to the number of translators/consultants we could capture in this study. Only a few, ~2.5% of the total responses, declared their role as business (and innovation) developers. These results are evidence that some participants could be identified as more closely related to other FAs within the EMMC, ultimately showing that all FAs are connected to the ones surveyed here.

**Figure 5.**
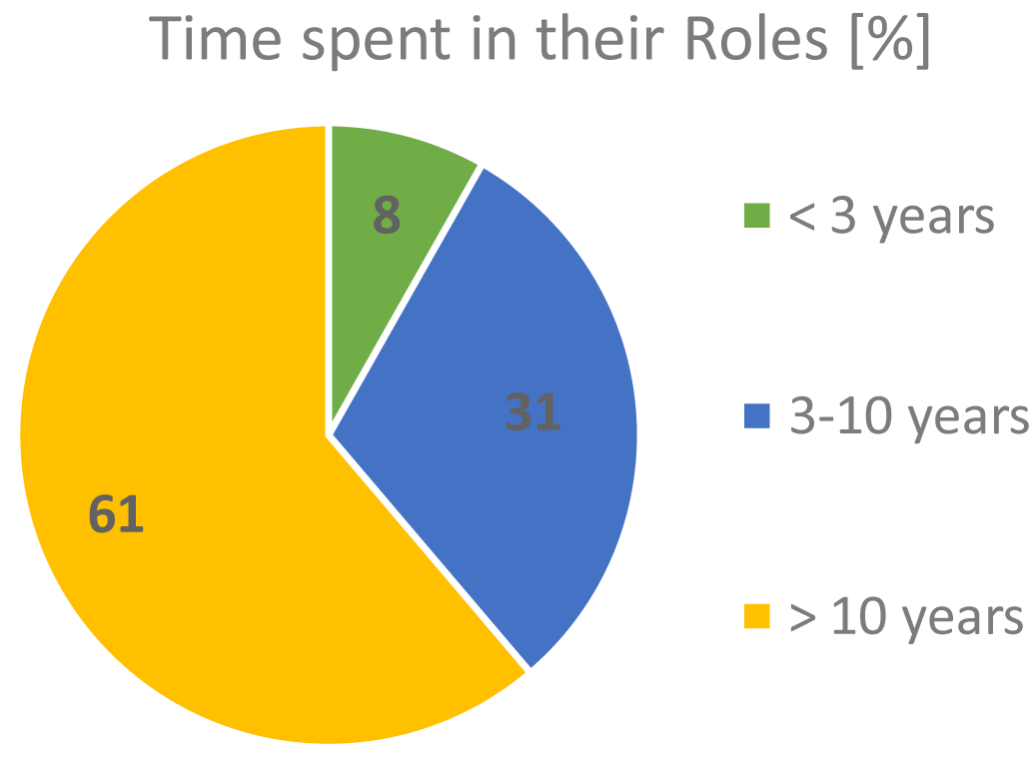
TIME RESPONDENTS SPENT PERFORMING THEIR ROLES.

The majority of our respondents (61.2%) had been in their roles for more than 10 years, while only 8.2% were relatively new to their activities, as shown in Figure 5. This gives high confidence and relevance of the outcomes of this survey to the FAs’ themes of interest.

The respondents were asked to share what types of research they are involved in within their organisations. The results, summarised in Table 2, show that the majority (~65%) are involved in the computationally and methodology driven environments of the two FAs surveyed. However, there are significant fractions of experimentally assisted (~15%) and data driven (~13%) modellers. It is also relevant to note that a non-negligible fraction (6%) of respondents are associated with digitalisation and interoperability aspects of modelling.

**Table 2.** T he types of research respondents perform in their organisation (HPC - high performance computing, HTC - high throughput computing, AI - artificial intelligence, ML - machine learning).

Type of research	Number of times selected	%
**Computationally driven (HPC modelling)**	77	37.9
**Method, algorithms and software development**	55	27.1
**Experimentally driven (assisted by modelling)**	31	15.3
**Data driven (AI/ML, HTC modelling)**	26	12.8
**Digitalisation and interoperability driven (interscale and ** **workflows)**	13	6.4
**Mathematical models**	1	0.5
**Total times selected**	**203**	

### Model scales and purposes of modelling

**Figure 6.**
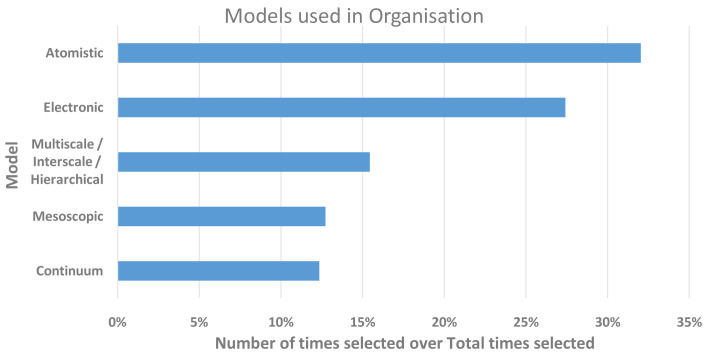
LEVELS OF MODELS USED IN RESEARCH WITHIN THE RESPONDENTS’ ORGANISATIONS. THE Y-AXIS SHOWS THE MODEL SCALE AND THE X-AXIS THE NUMBER OF TIMES A LEVEL WAS SELECTED. THE RESPONDENTS WERE ALLOWED TO SELECT MORE THAN ONE.

Figure 6 summarises the responses for the levels of models used by the respondents’ institutions. Given the large proportion of respondents based in academia, it is not surprising that atomistic and electronic scales dominated over mesoscopic and continuum scales, which are known to be more popular with industry and hence more often commercially applied and exploited. However, it is also clear that a significant proportion of the respondents’ institutions carried out research where scales were coupled, which also links to the FA for interoperability.

**Figure 7.**
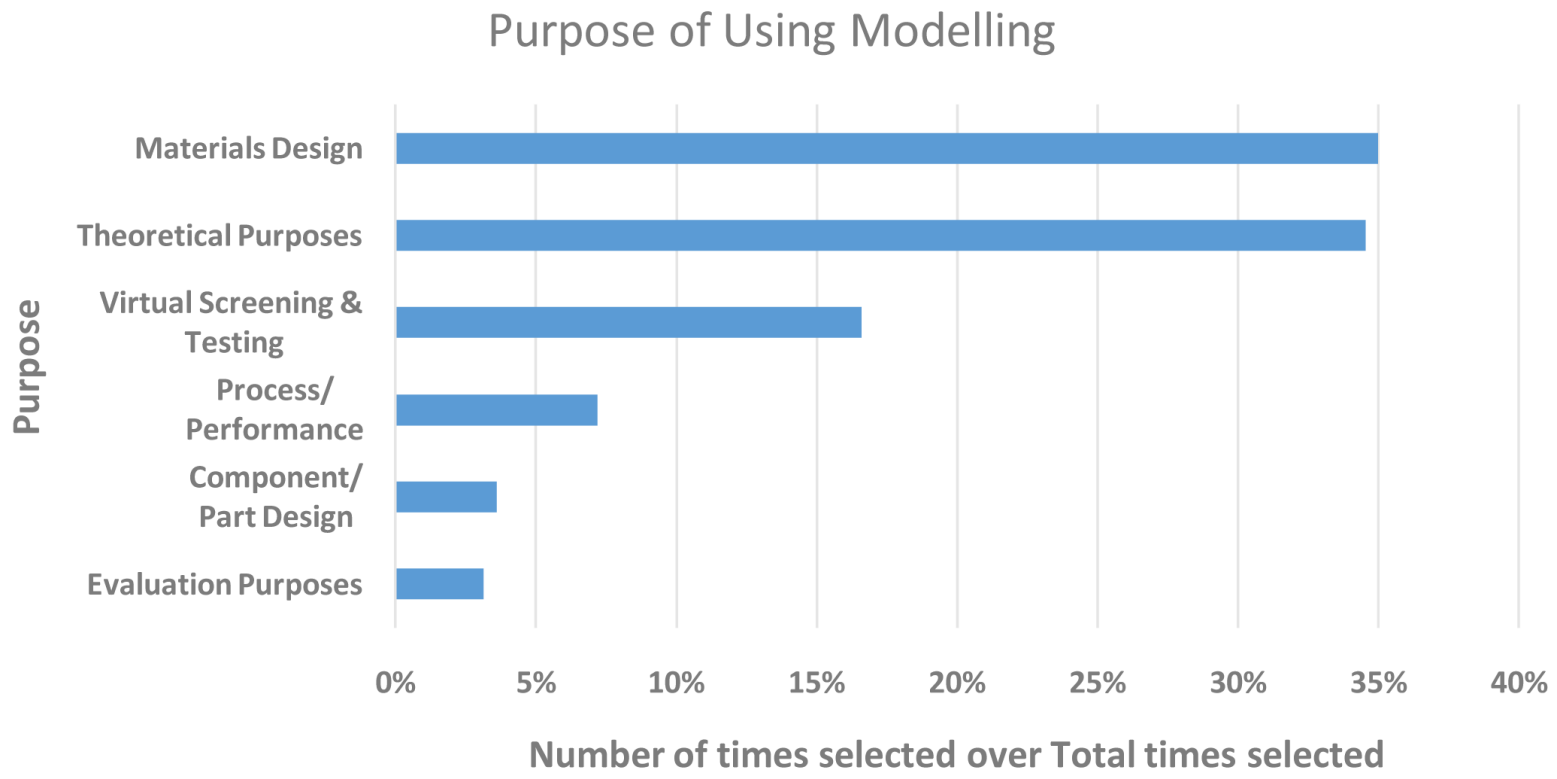
MAIN PURPOSES OF RESEARCH WITHIN THE RESPONDENTS’ ORGANISATIONS. THE Y-AXIS SHOWS THE PURPOSE AND THE X-AXIS THE NUMBER OF TIMES IT WAS SELECTED. THE RESPONDENTS WERE ALLOWED TO SELECT MORE THAN ONE.

Figure 7 clearly shows the main purposes for which research was carried out. The results were aligned with the types of work the respondents based predominantly in academia (~58%) and RTO (~25%) carry out; rating materials design and theoretical purposes as the main drivers, followed by virtual screening and testing. More industrially relevant purposes, such as process performance, component design and evaluation, scored comparatively low, which was unsurprising given the smaller fraction of industrially based respondents (~13%).

The respondents were asked to share the types of materials their modelling research lines focused upon. Table 3 summarises the responses scoring more than 4% of the total selections over all material types. The respondents were allowed to select multiple types and add new materials they considered specific to their research lines, hence the dominance of these in the “Other” category. The general trend seemed to be an equal, 50/50, split between solid state and soft matter, with a small fraction (~4%) of meta-materials.

**Table 3.** T he type of research respondents perform in their organisation.

Type of Material (sorted by number of times selected)	Number of times selected	%
**Metals**	54	17.2
**Fluids and gasses**	37	11.8
**Bio, medical and organic chemistry**	33	10.5
**Polymers**	33	10.5
**Ceramics**	30	9.6
**Glasses**	24	7.6
**Additively manufactured meta materials**	17	5.4
**Hydro-carbon solutions and tar**	13	4.1
**Other more specialised categories**	57	18.2
**Total times selected**	**314**	

### The future of modelling

The most important part of the survey tried to capture the perceived *modus operandi* of how modelling had changed in the past 5 years, shown in Table 4, and what the expectations of the surveyed audience were for the next 5 years, given in Table 5.

**Table 4.** C hanges of modelling trends and practices over the last 5 years (AI - artificial intelligence, ML - machine learning, NN - neural networks, GPU - graphical processing unit).

The changes over the last 5 years were …	Number of times selected	%
**None**	46	49.5
**Complex/multiscale modelling and workflows**	13	14.0
**The raise of databased modelling, ML, AI**	12	12.9
**More emphasis on data/post processing (scripting,** **automatisation, AI, ML, NN)**	7	7.5
**Better model/software quality**	7	7.5
**More computer power (GPUs, etc.)**	4	4.3
**Better accuracy of models**	2	2.2
**Models more time effective/better scaling of algorithms**	1	1.1
**Cloud computing**	1	1.1
**Total times selected**	**93**	

**Table 5.** C hanges of modelling trends and practices over the next 5 years (GPU - graphical processing unit).

The changes in the next 5 years will be …	Number of times selected	%
**More databased modelling, ML, AI**	28	28.3
**None**	21	21.2
**More complex/multiscale modelling and workflows ** **(interoperability)**	13	13.1
**More data driven modelling**	12	12.1
**More advanced software/workflows/automatisation**	11	11.1
**Better hardware (GPUs, advanced architectures, quantum ** **computing)**	7	7.1
**More cloud-based computing**	4	4.0
**Models more time effective/better scaling of algorithms**	2	2.0
**Better accuracy of models**	1	1.0
**Total times selected**	**99**	

It is clear from Table 4 that nearly half of the respondents had experienced no big changes in the type of modelling work carried out, nor the tools used, in the past 5 years. This is not entirely surprising given the identification make-up of the respondents in Table 1. Of the rest of the respondents, 14% had adopted and invested in workflow and multiscale technologies. A similar proportion (13%) had ventured into data driven modelling, with around 8% putting more emphasis on this area than on traditional modelling. Coincidentally, the same proportion fits the Digitalisation and interoperability driven (interscale and workflows) in Table 2. Only 8% acknowledged adoption of better models and software, and 4% exploited the benefits brought by increased HPC power. The remainder confirms that the smallest changes were seen in developments of accuracy and scalability performance of models as well as in the use of cloud computing.

The forward look in Table 5 is quite different. The only similarities with previous changes were the smallest expectations placed on the development of models’ accuracy and scalability performance, which are possibly the most difficult to achieve and require significant labour investment. The expectations of using cloud computing rose slightly (4%), as did those for increasing computer power (7%), which now included quantum computing.

The biggest changes in the forward outlook are in the distribution at the top. The number of responses of no change nearly halved (from 50% to 28%), while databased modelling using ML and AI nearly doubled (13% to 28%). The expectations for multiscale modelling and workflows stayed constant (13%), although these now also included an accent on interoperability. What also is considered a change is the establishment of data driven modelling (12%, which includes both physical equation based modelling as well as characterisation driven experiments) as commonplace. Furthermore, expectations were placed on sophisticated infrastructures via more advanced software, workflows and automatisation (11%).

### Software adoption factors

**Figure 8.**
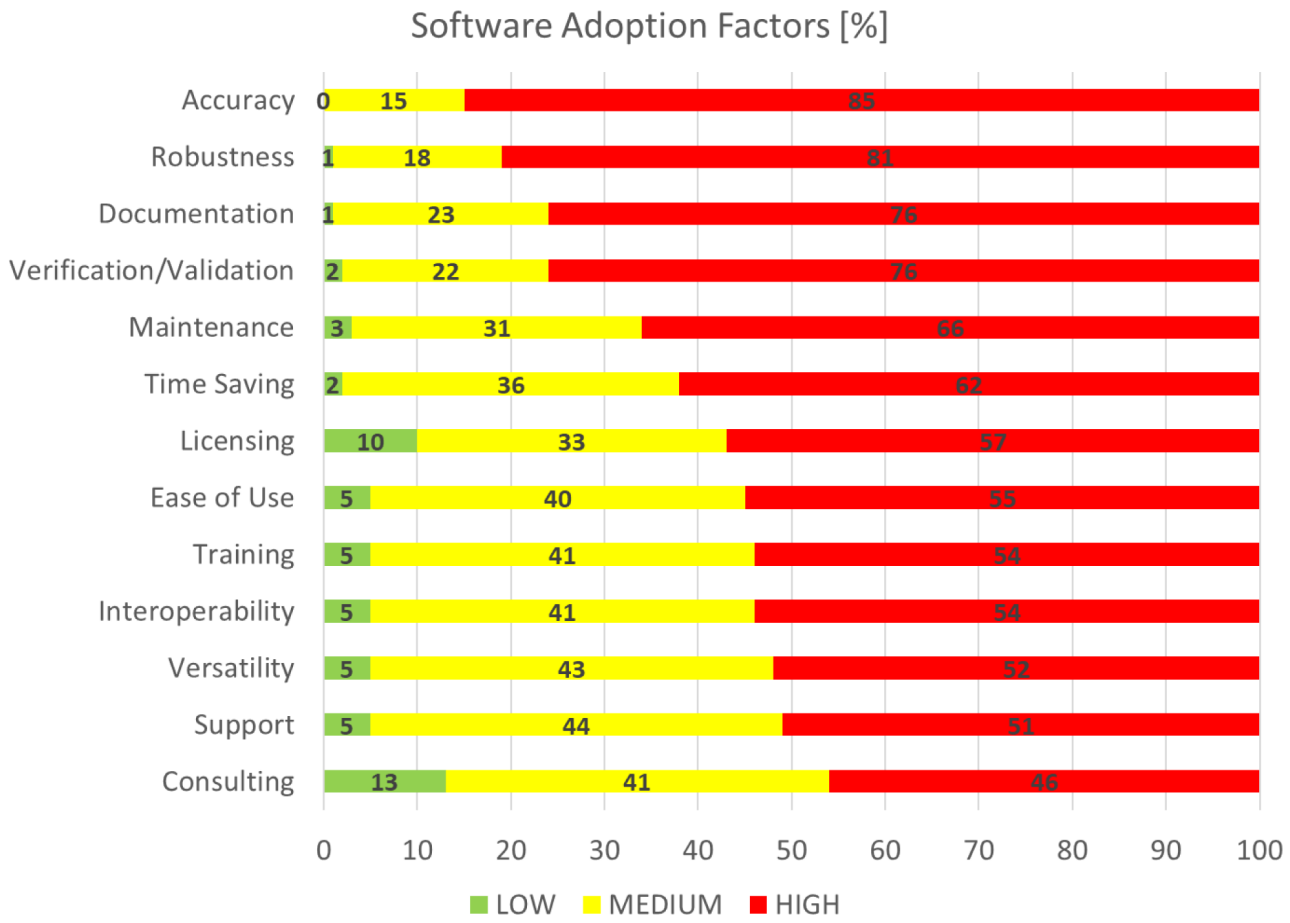
SOFTWARE ADOPTION FACTORS.

This section is concerned with the identification and rating of the factors deemed as important for the adoption of research software, which is of particular interest to the EMMC’s Software FA. Figure 8, software adoption factors, shows the aggregated performance of all factors that we asked the respondents to grade on a scale from 1 (least important) to 10 (most important). For visualisation purposes the rankings are grouped into three categories: the low (1–3), medium (4–7) and high (7–10) importance bands. The most important factor is clearly accuracy, while the least important is consulting; this is not surprising given the domination of academic and RTO profiles of the sampled audience in Figure 1. If we define top factors as those with responses where more than three quarters (¾) of them are high, then what defines success for research software engineers (RSE) and can be considered as of utmost importance to the EMMC’s Software FA are *accuracy*, *robustness*, *documentation* and *verification/validation*. Further analysis on the data here is better coupled with the outcomes in the next section.

### Software proliferation bottlenecks

**Figure 9.**
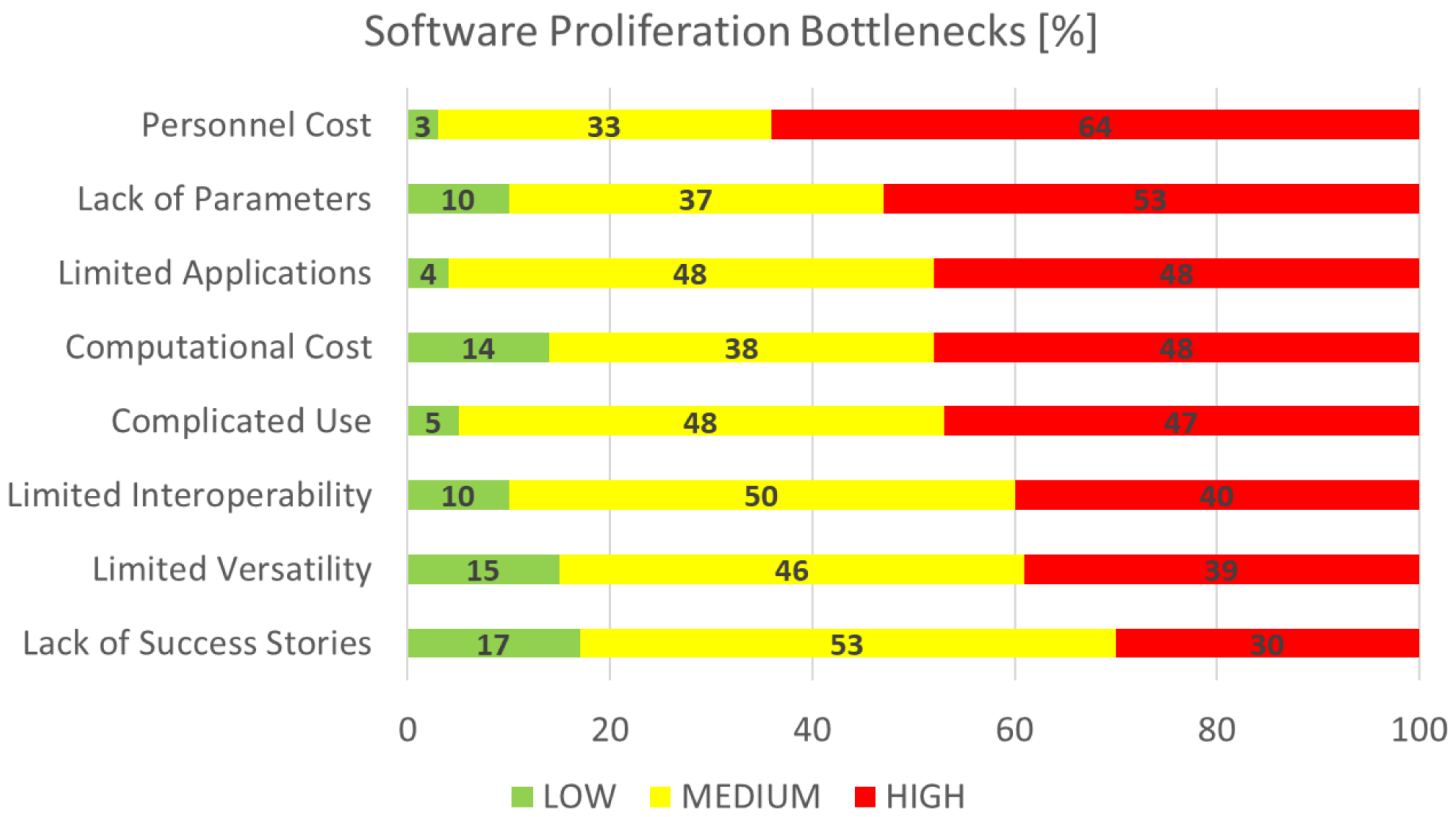
SOFTWARE PROLIFERATION BOTTLENECKS.

This section is concerned with the identification and rating of the factors that are deemed as bottlenecks for the wider spread and utilisation of research software, again of particular interest to the EMMC’s Software FA. Figure 9 shows the aggregated performance of all bottlenecks that we asked the respondents to grade on a scale from 1 (least important) to 10 (most important). For visualisation purposes the rankings are grouped into three categories: the low (1–3), medium (4–7) and high (7–10) importance bands. The most limiting factor is clearly *personnel cost,* while the least limiting is *lack of success stories*. The former is quite intriguing and possibly points towards the emerging trends of using software as a service (SaaS) rather than setting up and starting a new RSE group with an institution. At the present, the latter ( Usher *et al.*, 2020) is assumed as a strategic consideration by many research organisations; however, in some cases such groups can be split over geographically neighbouring institutions as the home institution subcontracts the services to the others.

As in the previous section, if we define top factors as those with responses where more than half (½) are high, then the major bottlenecks to sustaining success for research software are *Personnel Cost* and *Lack of Parameters*. These are closely followed by other important areas. *Limited applications* points towards further requirements for versatility, such as software suites providing for interoperable and/or multiscale workflows with possible data driven orchestration. *Computational costs* refers to the efficient utilisation of modern architectures (hybridised with accelerators) and programming paradigms (including modernisation of computer languages and extensions for accelerated parallelism). *Complicated use* refers to the cost of production of documentation (at all levels) and the delivery of targeted training (learning prerequisites for new users). Last but not least, we must acknowledge that all bottlenecks have the *personnel cost* dimension in common.

### New methodology adoption incentives

**Figure 10.**
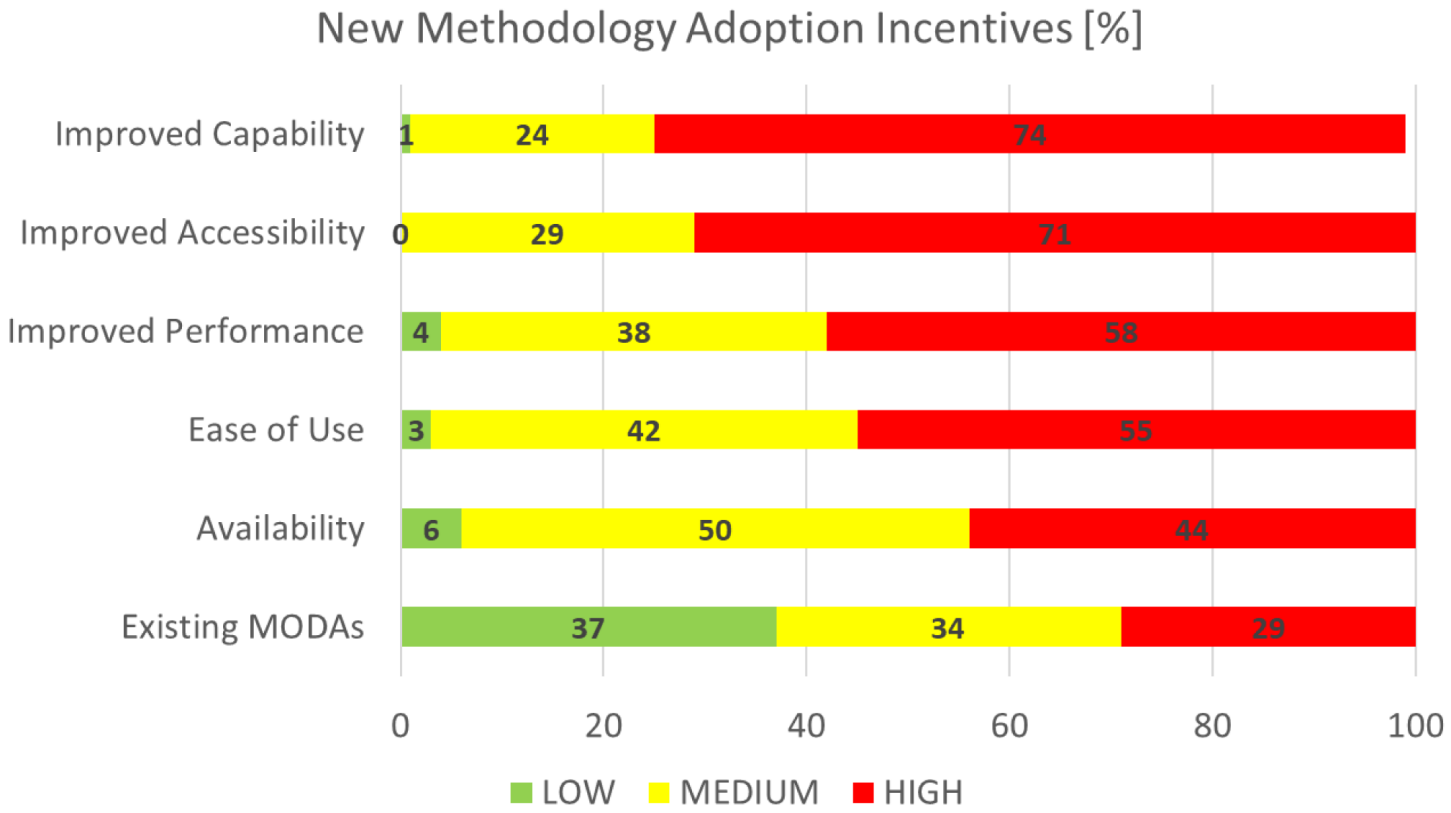
NEW METHODOLOGY ADOPTION INCENTIVES (MODA - MODELLING DATA [ DE BAAS, 2017]).

This section is concerned with the identification and rating of the incentives deemed as important for the adoption of new methodology, which is of particular interest to the EMMC’s Method Development FA. Figure 10 shows the aggregated performance of all incentives that we asked the respondents to grade on a scale from 1 (least important) to 10 (most important). For visualisation purposes the rankings are grouped in three categories: the low (1–3), medium (4–7) and high (7–10) importance bands. The most important factor is clearly *improved capability* and the least important *existing MODAs*. The latter is surprising; we believe it is actually connected to adoption by the user community.

### New methodology adoption deterrence

**Figure 11.**
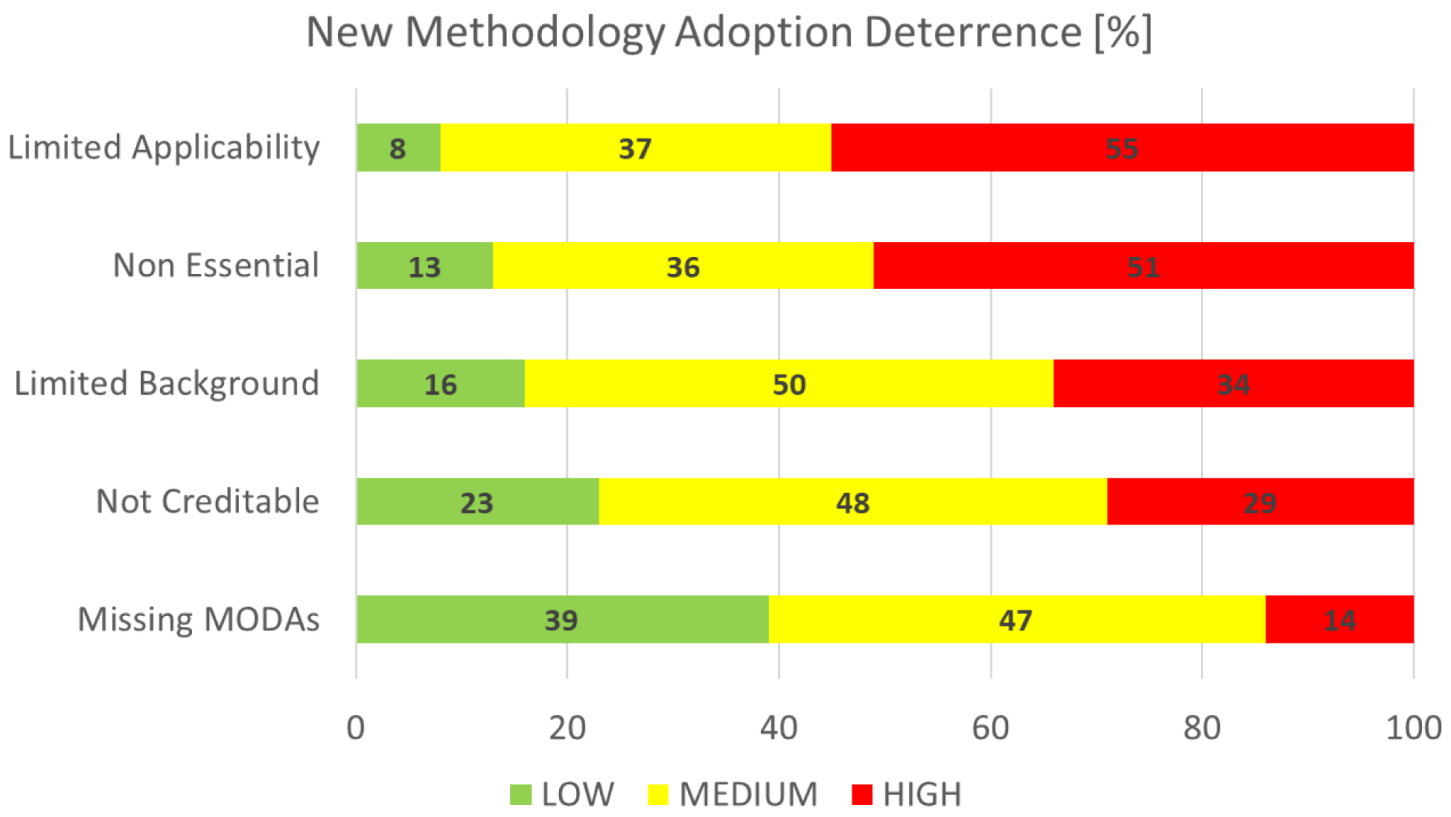
NEW METHODOLOGY ADOPTION DETERRENCE (MODA - MODELLING DATA [ DE BAAS, 2017]).

This section is concerned with the identification and rating of the factors that are deemed as bottlenecks for the adoption of new methodology, again of particular interest to the EMMC’s Method Development FA. Figure 11 shows the aggregated performance of all deterrence factors that we asked the respondents to grade on a scale from 1 (least important) to 10 (most important). For visualisation purposes the rankings are grouped in three categories: the low (1-3), medium (4-7) and high (7-10) importance bands. The biggest bottleneck clearly is *limited applicability,* while the smallest one is *missing MODAs*. The top bottleneck is understood to originate from new methods being more narrowly specific as opposed to being general purpose. However, the second factor, *non essential*, suggests that general purpose methods are somewhat more easily adopted and applied, and sufficient for the bulk of current modelling and simulation research.

### Properties and digital twins

***Properties prediction.*** We asked the respondents for the most important materials properties that their research aimed to measure and predict by using computer simulations or by addressing via developing materials modelling methodologies (methods, models, workflows, software). The respondents were allowed to select multiple types and add new ones they considered specific to their research lines.

Table 6 summarises the responses, which may be split roughly into three main areas of almost equal interest:

- Chemical: *chemical* and *thermodynamic* properties, scoring 34.6% of the total interest
- Physical: *electrical, optical* and *magnetic* properties, scoring 32.2% of the total interest
- Engineering: a collection of mechanical, fluidic and interfacial properties, scoring 33.2% of the total interest.

**Table 6.** M ost important materials properties to obtain via modelling and simulation.

Properties	Number of times selected	%
**Chemical**	67	17.3
**Thermodynamic**	67	17.3
**Electrical**	54	13.9
**Optical**	42	10.8
**Magnetic**	29	7.5
**Mechanical, elastic – stiffness**	29	7.5
**Mechanical, elastic – strength**	29	7.5
**Liquid-Vapour related**	14	3.6
**Rheological – adhesion**	13	3.4
**Mechanical, elastic – fatigue**	12	3.1
**Rheological – wetting**	12	3.1
**Mechanical, elastic - progressive damage**	10	2.6
**Mechanical, elastic – damping**	5	1.3
**Total times selected**	**388**	

***Digital twins.*** The survey asked “ *In the context of the comprehensive digital twin, with which disciplines should Materials Modelling software be integrated more closely?*”. The question offered two, non-mutually exclusive options and an optional comments section to provide further detail on the respondents’ choices.

The majority of respondents (85%) chose *experimental physical testing data (e.g., parameter identification process) and data management*. Only 13% chose *the manufacturing process (e.g., build processors for agile manufacturing)*, while 2% chose both along with a representative comment: “ *Experimental physical testing data is the natural discipline, but if we achieve a proper and accurate multiscale protocol Materials modelling can have a huge potential in the manufacturing for materials and drug design.*”

### How can the EMMC ASBL be of help?

This section is concerned with the open part of the survey asking for discussions on how the EMMC could help incentivise further adoption of digitalisation and best practices for modelling and simulation. The aggregated data from the respondents’ feedback and recommendations following up from this question are summarised as follows:

- Offer information, well-structured and easy to find
  - Case studies (advanced, liked to IP, an actual product, …)
  - Demonstrator cases
  - Link to organisations who may be able to contribute beyond EMMC’s offerings
  - Link to funding bodies who could enable “better materials modelling software”

- Offer training on best practises
  - Master Classes
  - Workshops
  - Tutorials
  - EU-level training with other organisations

- Have an active dialogue with all stakeholders (materials modelling users and developers)
  - Reach out to key stakeholders – engage leaders/influencers in their respective fields
  - Enable matchmaking of experts; collaboration with other organisations ( European Materials Characterisation Council [EMCC], International Association for the Engineering Modelling, Analysis and Simulation Community [NAFEMS]); formation of consortia
  - Join and track infrastructure projects

- Have a vision on where digitalisation is actually going
  - What are the challenges
  - Which gaps to close

- Support both free & open source software (FOSS) as well as commercial software
- Work on standardisation of software and file formats
- Work on roadmaps

## Conclusions

The EMMC focus areas Model Development and Software lead the EMMC’s activities in their domains and the key needs of their representative researcher communities are summarised as follows. For Software these were defined as more accurate, robust, well-documented and validated/verified software, with better availability of parameters data (easy generation of input), applicability (general purpose/demonstrators), scalable performance & lower complexity to use. For Model Development these were defined as improved capability, accessibility and performance of methods, with better applicability (general purpose over specificity).

This survey endorsed the point that digital tools drive the future of materials in chemical and manufacturing industries, by providing agility and speed in the development process. The materials digitalisation wave relies on using physics-based modelling approaches as well as data-driven approaches and their use together, to accurately predict and optimise industrial products in an early design stage. The materials research community, including methodology developers and software owners, provides cutting edge materials modelling methods and software that both require continued support by funding bodies and organisations, nationally and internationally.

## Data availability

eData: EMMC Software and Modelling Questionnaire. http://dx.doi.org/10.5286/edata/754 [7]

This project contains the following underlying data:

Software and Modelling Questionnaire_v2.xlsx (the data file is in a MicroSoft ^®^ Excel format and contains the anonymised raw data from the survey form as well as in separate data sheets the extracted and aggregated data and the data operations from which the tables and figures in this study were produced).

Data are available under the terms of the Creative Commons Attribution 4.0 International license (CC-BY 4.0).

## Ethics and consent

The survey and the analysis published within this paper have been endorsed, supported and approved by EMMC BoD, the Senior Leadership Team of the Scientific Computing Department at STFC and the VIMMP, OntoCommons and DOME 4.0 consortia. This research supports the transparency of the needs and expectations of the communities that EMMC, STFC, VIMMP, OntoCommons and DOME 4.0 represent and support. The EMMC BoD consider this study exempt from ethical or consent considerations. There is no personal or sensitive data collected for the survey.
