# Supplementary material for: Evolving requirements for materials modelling software and underlying method developments: an inventory and future outlook
Source: Open Res Eur. 2022 Aug 1;2:86. Originally published 2022 Jul 7. [Version 2] doi: 10.12688/openreseurope.14843.2 (PMC10446040; doi:10.12688/openreseurope.14843.2)
Supplement: Peer review history

# Peer review: Reviewer response for version 2

**DOI:** 10.21956/openreseurope.16223.r29818

**Published:** 2022-08-12

**Authors:** Erich Wimmer

**Affiliations:** 1 Materials Design SARL, Montrouge, France

The revised version reads well. At the very beginning of the abstract, the author makes a reference to "the survey". It might be helpful to state which survey is meant.

Is the study design appropriate and does the work have academic merit?

Yes

Is the work clearly and accurately presented and does it cite the current literature?

Yes

If applicable, is the statistical analysis and its interpretation appropriate?

Partly

Are all the source data underlying the results available to ensure full reproducibility?

Yes

Are the conclusions drawn adequately supported by the results?

Yes

Are sufficient details of methods and analysis provided to allow replication by others?

Yes

Reviewer Expertise:

Computational materials science and industrial applications.

I confirm that I have read this submission and believe that I have an appropriate level of expertise to confirm that it is of an acceptable scientific standard.

# Peer review: Reviewer response for version 2

**DOI:** 10.21956/openreseurope.16223.r29817

**Published:** 2022-08-02

**Authors:** Costas A. Charitidis

**Affiliations:** 1 Research Lab of Advanced, Composite, Nano-Materials and Nanotechnology, Materials Science and Engineering Department, School of Chemical Engineering, National Technical University of Athens, Athens, Greece

No further comments.

Is the study design appropriate and does the work have academic merit?

Yes

Is the work clearly and accurately presented and does it cite the current literature?

Yes

If applicable, is the statistical analysis and its interpretation appropriate?

Yes

Are all the source data underlying the results available to ensure full reproducibility?

Yes

Are the conclusions drawn adequately supported by the results?

Yes

Are sufficient details of methods and analysis provided to allow replication by others?

Yes

Reviewer Expertise:

Material Science and Computational Material Science

I confirm that I have read this submission and believe that I have an appropriate level of expertise to confirm that it is of an acceptable scientific standard.

# Peer review: Reviewer response for version 1

**DOI:** 10.21956/openreseurope.16033.r29658

**Published:** 2022-07-26

**Authors:** Erich Wimmer

**Affiliations:** 1 Materials Design SARL, Montrouge, France

This is a very useful document and the author deserves credit for having carried out this work.

 This work addresses a wide range of issues related to materials modelling and hence provides a valuable view of the current status. For example, it is interesting to see the cost of personnel as a major limiting factor. The need for accuracy and "easy generation of input" also stand out as key bottlenecks. Perhaps the author could expand on the latter. Is it the lack of, for example, forcefield parameters? Is it too difficult to create models, e.g. crosslinked polymers? Is it something else?

 My major concern is the exceedingly small number of industrial participants and the resulting strong bias towards RTA's and academic researchers. This fact is clearly not due to a lack of effort by the author, but rather reflects the still very low use of materials modelling in industry. It would be useful to provide a more detailed analysis of this fact in this work. Is the gap too wide between actual industrial requirements and current capabilities of (discrete) materials modelling? Are there other factors such as a lack of skilled industrial modelers?

 Overall, this is a very useful piece of work and I recommend its indexing.

Is the study design appropriate and does the work have academic merit?

Yes

Is the work clearly and accurately presented and does it cite the current literature?

Yes

If applicable, is the statistical analysis and its interpretation appropriate?

Partly

Are all the source data underlying the results available to ensure full reproducibility?

Yes

Are the conclusions drawn adequately supported by the results?

Yes

Are sufficient details of methods and analysis provided to allow replication by others?

Yes

Reviewer Expertise:

Computational materials science and industrial applications.

I confirm that I have read this submission and believe that I have an appropriate level of expertise to confirm that it is of an acceptable scientific standard.

# Peer review: Reviewer response for version 1

**DOI:** 10.21956/openreseurope.16033.r29659

**Published:** 2022-07-19

**Authors:** Costas A. Charitidis

**Affiliations:** 1 Research Lab of Advanced, Composite, Nano-Materials and Nanotechnology, Materials Science and Engineering Department, School of Chemical Engineering, National Technical University of Athens, Athens, Greece

The manuscript entitled “Evolving requirements for materials modelling software and underlying method developments: an inventory and future outlook” reports the results of a survey on material modelling. The survey was aimed towards members of the material modelling community, and is successful in mapping the tendencies, bottlenecks, and potential future developments in this area of interest. The manuscript is well written and structured, while the results and methods are well explained. Therefore, my suggestion is that the article is approved. A few points can be addressed to improve the article.

1. The plain language summary is exactly the same as the conclusion. It is rather suggested that the conclusions may include points that can be taken out from the survey results.
2. In the “Results” Section, under the Subsection “The Players”, it is mentioned that 58.2% of the survey participants are based in Academia but within the subsection (and the corresponding Figures and Tables) there is no mention or discussion about the findings for Academia participants.
3. In page 4, it is written that “the majority of our respondents were using or developing software (~56%) and 23 of them were in a managerial role (~12% of the total responses).”. Since the total number of participants were 98, 23 respondents cannot consist of the 12% of the total responses. Please explain this small inconsistency.
4. In page 5, in the 5 ^th^ line of the paragraph at the left column (and at Figure 7), one potential choice of the survey concerning the main research purposes is “theoretical purposes”. What it is denoted by this term?
5. The percentages in tables 3 and 6 don’t sum up to 100%, which could create confusion.
6. The Table 4 which is discussed under the Subsection “The future of modelling” might be also correlated with Table 2 and not only with Table 1. For example, it is mentioned that “A similar proportion (13%) had ventured into data driven modelling, […]” which fits the percentage provided in Table 2 (12,8%).
7. In page 8, in the paragraph at the right column, it is mentioned that “the number of responses of no change nearly halved (from 50% to 28%), while data driven modelling nearly doubled (13% to 28%).” Three lines later, it is written “These were closely followed by data driven modelling (12%) […]”. The data driven modelling cannot be 28% and 12% at the same time, in the first phrase the term “data driven modelling” should be replaced by the term databased modelling including ML and AI to avoid confusion.
8. In Figures 8 and 9, for software adoption factors and software proliferation bottlenecks, respectively, how is it possible to adopt a software due to its versatility (52/43/5, high/medium/low importance, respectively) and at the same time claim that the software is for limited applications?
9. Where the term “Lack of parameters” shown in Figure 9 is referring to?
10. Since the biggest bottleneck for software proliferation is the personnel cost according to the findings of the survey, how it is suggested to overcome this bottleneck and what can be potentially the role of EMMC on that?

Is the study design appropriate and does the work have academic merit?

Yes

Is the work clearly and accurately presented and does it cite the current literature?

Yes

If applicable, is the statistical analysis and its interpretation appropriate?

Yes

Are all the source data underlying the results available to ensure full reproducibility?

Yes

Are the conclusions drawn adequately supported by the results?

Yes

Are sufficient details of methods and analysis provided to allow replication by others?

Yes

Reviewer Expertise:

Material Science and Computational Material Science

I confirm that I have read this submission and believe that I have an appropriate level of expertise to confirm that it is of an acceptable scientific standard.

# Response

**Published:** 2022-07-27

**Authors:** Ilian Todorov

**Affiliations:** UKRI STFC, UK

Dear Costas Charitidis, Thank you for reading carefully the paper! Your engagement with its review is exemplary and has generated useful comments to improve it!

**Q1.** The plain language summary is exactly the same as the conclusion. It is rather suggested that the conclusions may include points that can be taken out from the survey results.

**A1:** It is the practice of this journal to be upmost informative. Hence, the initial summary, acting as a comprehensive abstract, is a concise version of the conclusions.

**Q2.** In the “Results” Section, under the Subsection “The Players”, it is mentioned that 58.2% of the survey participants are based in Academia but within the subsection (and the corresponding Figures and Tables) there is no mention or discussion about the findings for Academia participants.

**A2:** We have not tracked the difference between academic and non-academic respondents on the survey results. However, we have commented at places on particulars about the large representation of academic respondents.

**Q3:** In page 4, it is written that “the majority of our respondents were using or developing software (~56%) and 23 of them were in a managerial role (~12% of the total responses).”. Since the total number of participants were 98, 23 respondents cannot consist of the 12% of the total responses. Please explain this small inconsistency.

**A3:** As written in the text "The respondents were asked to share which roles they take

 on in their respective organisations, which are shown in Table 1." and in Figure 4's caption "THE 98 RESPONDENTS WERE EACH ALLOWED TO SELECT MORE THAN ONE ROLE.".  Respondents are allowed to have more than one role within their selected organisation. Hence percentages are no longer weighted to unity and their sum will not give 100%. I'll strive to include "(multiple choice allowed)" in the text.

**Q4:** In page 5, in the 5th line of the paragraph at the left column (and at Figure 7), one potential choice of the survey concerning the main research purposes is “theoretical purposes”. What it is denoted by this term?

**A4:** Theoretical purposes is often referred as basic study or research, that are often regarded as fundamental and also not yet experimentally accessible or relevant.

**Q5:** The percentages in tables 3 and 6 don’t sum up to 100%, which could create confusion. 

**A5:** This is related to the Q3 and answered in A3.

**Q6:** The Table 4 which is discussed under the Subsection “The future of modelling” might be also correlated with Table 2 and not only with Table 1. For example, it is mentioned that “A similar proportion (13%) had ventured into data driven modelling, […]” which fits the percentage provided in Table 2 (12,8%).

**A6:** That is a useful observation although there is no per se direct correlation between the two data sets as the former is an integrated change of research change observations over 5 years whereas the other set(s) static view of respondents' attribution to a particular role. It is true that both describe the emergence of multiscale workflows.

**Q7:** In page 8, in the paragraph at the right column, it is mentioned that “the number of responses of no change nearly halved (from 50% to 28%), while data driven modelling nearly doubled (13% to 28%).” Three lines later, it is written “These were closely followed by data driven modelling (12%) […]”. The data driven modelling cannot be 28% and 12% at the same time, in the first phrase the term “data driven modelling” should be replaced by the term databased modelling including ML and AI to avoid confusion.

**A7:** You are absolutely correct here as there is indeed a confusion brought up by the wording.  The paragraph will change as: "The biggest changes in the forward outlook are in the distribution at the top. The number of responses of no change nearly halved (from 50% to 28%), whereas the databased modelling using ML and AI have doubled (13% to 28%).  The expectations for multiscale modelling and workflows stayed constant (13%), although this category now also included an accent on interoperability.  What also is considered a change is the establishment of data driven modelling (12%, which includes both physical equation based modelling as well as characterisation driven experiments) as commonplace.  Furthermore, expectations are placed on sophisticated infrastructures via more advanced software, workflows and automatisation (11%)." It would be nice to make Table 4 and 5 are of the same dimensions in the print. Going over the captions I have also detected that some double full stops have occurred, which also needs correcting.

**Q8:** In Figures 8 and 9, for software adoption factors and software proliferation bottlenecks, respectively, how is it possible to adopt a software due to its versatility (52/43/5, high/medium/low importance, respectively) and at the same time claim that the software is for limited applications?

**A8:** If software is adopted then it does the job well for what it is needed and versatility is possibly not so essential and relevant as it does not score high - it is at the bottom of the scale. However, one would adopt a software solution that is versatile to offer a number of solutions/options/methods/workflows under its hood, i.e. quantum (QM) driver, atomistic (MM) driver, and mesoscopic (DPD/LB) scale driver. On the other hand "limited applications" does not refer to versatility directly but indirectly. It really refers to applicability of the solution to specific systems (as do DFT functionals) and the availability of benchmarks and use cases to demonstrate a more extended applicability (variety of models, model systems, functional, integration schemes/ensembles), beyond those driven by the software creators' research agenda.

**Q9:** Where the term “Lack of parameters” shown in Figure 9 is referring to?

**A9:** The general meaning of those is the material relation parameters for the specific physics equation coupling parameters. Hence, these refer to model parameters in general, which in the MD/DPD world are those of the force-field (interaction). DFTB it is also very similar.  The same considerations apply for mesoscopic scale (DPD/LB) and continuum software (FEM,CFD, etc.) which solve equations of state dependent on parameters that need to be supplied by the modeller.

**Q10:** Since the biggest bottleneck for software proliferation is the personnel cost according to the findings of the survey, how it is suggested to overcome this bottleneck and what can be potentially the role of EMMC on that?

**A10:** The paper was designed to show unbiased outcomes of the survey and not supposed to suggest solutions, especially as the one you are asking for. I can hazard many answers as I have pleaded to senior officials of funding bodies and in academia over the last 2 decades.  Hence, the existence of SSI (UK) and such like organisations world wide (RSSI in the USA) and RSE like associations (Society of RSE in the UK, WSSPE in USA, etc.)

 Kind Regards, Ilian Todorov
